# One Hundred Faces of Geraniol

**DOI:** 10.3390/molecules25143303

**Published:** 2020-07-21

**Authors:** Wanda Mączka, Katarzyna Wińska, Małgorzata Grabarczyk

**Affiliations:** Department of Chemistry, Wroclaw University of Environmental and Life Science, Norwida 25, 50-375 Wroclaw, Poland

**Keywords:** geraniol, bioactivity, antioxidant, anticancer, antimicrobial

## Abstract

Geraniol is a monoterpenic alcohol with a pleasant rose-like aroma, known as an important ingredient in many essential oils, and is used commercially as a fragrance compound in cosmetic and household products. However, geraniol has a number of biological activities, such as antioxidant and anti-inflammatory properties. In addition, numerous in vitro and in vivo studies have shown the activity of geraniol against prostate, bowel, liver, kidney and skin cancer. It can induce apoptosis and increase the expression of proapoptotic proteins. The synergy of this with other drugs may further increase the range of chemotherapeutic agents. The antibacterial activity of this compound was also observed on respiratory pathogens, skin and food-derived strains. This review discusses some of the most important uses of geraniol.

## 1. Introduction

**Figure 1:**
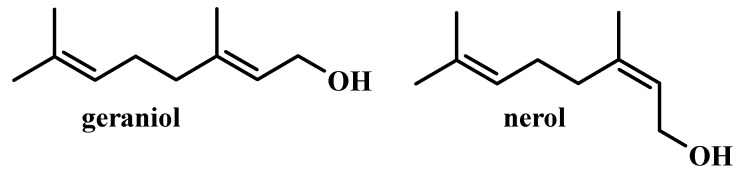
Chemical structure of geraniol and nerol.

Geraniol (Figure 1) is one of the most common fragrance ingredients in consumer products on the European market. It has a characteristic, well accepted among consumers, described as sweet, floral, rose-like, with a hint of citrus fruit. The presence of this compound was detected in 76% of the deodorants tested, 41% of detergents and cleaners and 33% of cosmetics based on natural ingredients [1]. At a concentration of 10 ppm, its taste is described as sweet-flowered rose, citrus-fruit with waxy nuances [2]. Pure geraniol is a clear or pale yellow oil that dissolves in most organic solvents but is not soluble in water. Geraniol is an important constituent of many essential oils, where it coexists with its nerol stereoisomer (Figure 1) and the products of geranial and neral oxidation [3].

So far, it has been found that this compound is present in more than 250 essential oils, including oil from *Monarda fistulosa* [4] (95%), ninde oil [5] (66.0%), rose oil [6] (44.4%), and palmarosa oil (53.5% and 80.9% geraniol in leaf) [7,8,9]. The essential oil is isolated from such plants as *Pelargonium graveolens*, *Cymbopogon martinii var motia* [10], *Cymbopogon winterianus Jowitt* syn. *Cymbopogon nardus* L., *Cymbopogon winterianus*, and *Cymbopogon jwarancusa* [11].

Table 1 (below) contains an overview of plants with a geraniol content of more than 18% in the volatile fraction.

The growing interest in geraniol has led to numerous reports on the use of biotechnological methods in the production of this compound in recent years. Researchers mainly use genetic engineering to modify microorganisms such as *Escherichia coli*, *Methanococcus maripaludis* or *Saccharomyces cerevisiae* in order to optimize geraniol production processes [36,37,38,39].

It was also noted that this compound has very interesting biological properties. Nowadays, the use of this compound as an antimicrobial agent [40], as a plant insect repellent [41], anti-inflammatory agent [42], anthelmintic [43]. It should not be forgotten that geraniol is a substrate in the synthesis of other compounds of health-promoting importance, such as vitamins A and E [44].

Taking into account the above information, in our work we focused on discussing research that used geraniol as a potential phytopharmaceutical drug with antimicrobial and anticancer properties.

## 2. Metabolism of Geraniol

To survive, every organism must have an elimination system of potentially harmful substances. The detoxification process allows for the conversion of lipid-soluble substances into water-soluble metabolites, which can be easily excreted from the body. Three phases can be distinguished in the detoxification system. In phase I of detoxification, the structure of the xenobiotic undergoes such an enzymatic modification (biotransformation) that it will not interact with lipophilic destinations. The observed reactions are mainly hydroxylation (most often catalysed by cytochrome P450 monoxygenases (CYP)) [45], oxidation (dehydrogenases) [46] or hydrolysis (carboxylesterase (CCE)) [47]. In the liver, in particular, members of CYP1, CYP2 and CYP3 families are involved in biotransformation of about 75% of all drugs used in humans [48]. In turn, phase II detoxification reactions usually consist of coupling phase I detoxification products with highly polar compounds such as sugars, amino acids, phosphates or glutathione. These reactions are catalyzed by transferases [49]. Phase III detoxification involves the transport of phase II conjugates from the cells for excretion or sequestration. The proteins involved in this phase include *p*-glycoproteins, multi-drug resistance proteins (MDR) and other ATP-binding cassette (ABC) transporters [47,50]

Although the first studies on geraniol metabolism in animals were undertaken as early as the 1980s, surprisingly little is known about it. Hydroxylation in the allyl position of geraniol seems to be a common reaction in animals, as it occurred not only in rats but also in insects [51] and was not observed in plants and microbial cultures [52]. In rats, apart from 8-hydroxygeraniol, 8-carboxygeraniol, (*2E*,6*E*)-3,7-dimethyl-octadiene-1,8-dioic acid, geranoic acid and 3-hydroxycitronelic acid were formed [53].

Since geraniol is present in many fragrance compositions used by the perfume industry, it is important to study its metabolism in skin cells. For this purpose, geraniol has been incubated with recombinant *E. coli* Tebu-bio bacteria containing human CYP bactosom (CYP1A1, 1B1, 2B6, 2E1, 3A5). The bacteria contained a CYP cocktail similar to skin cells, which provided a convenient method for testing skin metabolism using CYP isoforms found in the skin. CYP2B6 showed high activity in geraniol metabolism before CYP1A1 and CYP3A5. CYP1B1 and CYP2E1 showed low activity. Nevertheless, CYP1A1 and CYP3A5 are responsible for the majority of geraniol metabolism in the skin because they dominate in it [54].

In turn, CYP2B6 is the dominant isoform of CYP in the metabolism of xenobiotics in the liver [55]. In studies conducted in the human liver microsomal system, geraniol inhibited CYP2B6 activity. It is worth noting that CYP2B6 mediates in metabolism of some important drugs such as bupropion [56], cyclophosphamide [57], efavirenz [58], sibutramine [59] and tamoxifen [60]. Therefore, it is important to determine how it may affect the metabolism of medicines. It was found that it inhibited bupropion hydroxylation via CYP2B6 and showed stronger activity than the known CYP2B6 inhibitor—thio-TEPA [61]. Research suggests that geraniol is a competitive inhibitor of this enzyme. Considering that CYP2B6 plays a role in activation of pro-carcinogens such as aflatoxin B1 [55], cyclophosphamide and 4-(methylnitrosamino)-1-(3-pyridyl)-1-butanone (NNK), geraniol may be useful as a chemoprevention agent [54].

## 3. Geraniol Bioavailability

In order to simulate in vitro geraniol penetration through the intestinal barrier, tests have been conducted using the NCM460 cell line derived from primary cells of normal human colon mucosa. A high tendency of geraniol to penetrate through the monolayer of cells was found, and, additionally, it was indicated that geraniol can be actively transported from the intestinal lumen into the bloodstream and was not degraded in the digestive tract. Geraniol absorbed in plant fibres gave a total bioavailability of 16%, which indicates that the fibre has the ability to retain geraniol in the intestines, allowing it to reach the colon [62]. Surprisingly, after intravenous administration, geraniol is excreted from the bloodstream with a relatively short half-life of about 12 min, even at concentrations reaching 300 µg/mL The researchers explain this result by its binding to the cellular and protein components present in the blood and by its penetration into the lipid compartments of the body [48].

In studies carried out on Sprague-Dawley rats, the absolute availability of geraniol was determined at 92% in case of administration of geraniol emulsified in glycerol. The maximum blood concentration was found after 30 min and was about 270 µg/mL It is worth emphasizing that it was similar to that obtained after intravenous administration of the same dose of geraniol. Geraniol also easily overcomes the blood–brain barrier, but its concentration in the rat’s cerebrospinal fluid drops rapidly over time, similar to that of blood [48].

## 4. Antimicrobial Properties of Geraniol

An extensive review paper on the antimicrobial properties of geraniol has recently been published [63]. Such an activity of this compound may result mainly from its non-polar character, thanks to which it may disrupt the lipid structure of the microorganism’s cell membrane, interact with its components, making it more permeable also for other compounds, e.g., antibiotics. Geraniol can also penetrate the cell interior and limit the growth of the microorganism by binding in intracellular places of key importance for its survival [63].

Geraniol is a common component of many essential oils with antimicrobial properties [64]. The activity of 96 oils and separately 23 of their components against *Campylobacter jejuni*, *Escherichia coli*, *Listeria monocytogenes* and *Salmonella enterica* strains isolated from food and hospitals was tested [2,65]. It is estimated that more than half of all hospitalizations due to poisoning are related to the consumption of contaminated meat, since meat and meat products are an ideal nutritional matrix and are therefore susceptible to microbial contamination during the slaughter, processing and storage of food, which can promote the development of pathogenic microorganisms [66]. It was found that monoterpenic alcohols (linalol, nerol, citronellol, geraniol) had higher antibacterial than antifungal properties, with geraniol being most active against *E. coli* (BA_50_ = 0.15), *S. enterica* (BA_50_ = 0.15) and *L. monocytogenes* (BA_50_ = 0.28) [2,65]. In another study, the antibacterial activity of geraniol was also found against *Salmonella typhimurium* [2,67].

Geraniol also inhibited the growth of the enterotoxigenic strain of *E. coli* (ETEC), which is responsible for about 200 million diarrhea cases per year and causes about 380 thousand deaths. It is worth noting that, in developing countries, ETEC is the main health problem in children under 5 years of age [68].

However, a major limitation in the use of geraniol in the protection of food products is the hydrophobic nature of this compound, which makes it difficult to obtain its uniform dispersion in food products with high water content, resulting in a dramatic loss of geraniol activity. The preparation of geraniol in the form of nanoemulsions seems to be a way to solve this problem. The obtained nanoemulsion with Miglyol 812 was effective against *E. coli* K12 and *L. innocua* growing on a medium simulating meat. Geraniol was able to reduce the number of *E. coli* and *L. innocua* colony by about 3 logCFU/mL. *Pseudomonas lundensis* proved to be more resistant to nanoemulsion as a decrease of about 1.2 log CFU/mL was observed [66].

In a study conducted by Yegin et al., geraniol was trapped in amphiphilic triblock copolymer Pluronic^®^ F-127 nanoparticles [69]. Researchers obtained nanoparticles between 26 and 412 nm in size. In this form, geraniol was active against *E. coli* O157: H7 (ATCC 700728) and *S. enterica* serovar *Typhimurium* LT2 (ATCC 700720), which were developing on the surface of spinach. Nanoparticles with geraniol showed prolonged release within 24 h. Reductions of pathogens on treated spinach surfaces were from 0.3 to 4.2 log CFU/cm^2^. In another work, the same team examined the stability of products with geraniol nanoparticles stored at different temperatures. Geraniol nanoparticles reduced the amount of pathogens to an undetectable level (detection limit: 0.5 log CFU/cm^2^) in samples stored at 5 °C, 15 °C and, after 10 days of storage, at 25 °C. The use of encapsulated geraniol can therefore significantly reduce the risk of the development of pathogenic micro-organisms on the surface of spinach, preventing their transmission to consumers, thus helping to preserve the health of the products and their suitability for human consumption and nutrition [70].

Bhattamisra et al. [71] has studied the effects of geraniol in the treatment of gastric infections caused by *Helicobacter pylori.* This bacterium can result in gastritis and gastric or duodenal ulcers. In their study, ulcers were induced in rats by injecting acetic acid into the submucosal layer of the stomach, while bacteria were inoculated in the oral cavity for 1 week. Geraniol was used, together with the following drugs, to treat such infections: amoxicillin 50 mg/kg; clarithromycin 25 mg/kg, omeprazole 20 mg/kg and additionally geraniol (15 and 30 mg/kg). After 14 days, it was found that, in the group with geraniol, the ulcerative index was significantly reduced compared to the control group (4.13 ± 0.43). In addition, geraniol in an amount (30 mg/kg) added to the therapy resulted in a significant increase in gastric pH and a decrease in total acidity and gastric juice volume. Geraniol significantly weakened myeloperoxidase activity and increased the total glutathione level in the gastric mucosa. It was observed that geraniol exhibited significant anti-ulcer and anti-*H. pylori* activity in rodent model.

Geraniol administered in the aerosol inhibited the development of such dangerous human pathogens as: *Haemophilus influenzae*, *Streptococcus pneumoniae*, *S. pyogenes* and *Staphylocococcus aureus* [2]. It also inhibited the growth of *Cryptococcus neoformans*, which is responsible for the development of drug-resistant infections in the last stages of AIDS. Citronellol (minimum inhibitory concentration, MIC = 64 µg/mL), nerol (MIC = 128 µg/mL) and geraniol (MIC = 64 µg/mL) were also active in vitro against *Mycobacterium tuberculosis* which causing tuberculosis [2,72].

The unpleasant odour of human sweat occurs when skin secretions come into contact with the microflora on the skin. The interaction between the skin bacteria leads to the microbiological conversion of odourless apocrine sweat into odorous compounds [73]. The use of geraniol in antiperspirants as a growth inhibitor of microorganisms is limited due to its high volatility. In order to improve geraniol release stability, a study conducted by Nee et al. [74] attempted to encapsulate it in capsules using dextran as a carrier. The size of the obtained nanoparticles ranged from 70 to 110 nm, with an average size of 88 nm, and the capsulation yield reached 69.24%. Geraniol was completely trapped in the internal structure of the polymer matrix and 81.28% of geraniol was released from the nanoparticles within 48 h. The antimicrobial efficacy of the nanoparticles has been tested on human odour producing bacteria. In the diffusion test, both Gram-positive bacteria (*Staphylococcus epidermidis, S. hominis, Bacillus subtilis, B. cereus*) and Gram-negative bacteria (*Pseudomonas* sp.) were sensitive to geraniol in this form. In the case of *S. hominis,* the dependence of bactericidal properties of nanoparticles with geraniol on concentration was studied. At the minimum bactericidal concentration (MBC = 1.25 mg/mL) a 99.9% decrease was observed in relation to the control. [74]

The essential oil of *Cymbopogon martini*, the main ingredient of which is geraniol, was active against *S. cerevisiae*. Its action is a two-stage process. The first step is to passively penetrate the lipid membrane and disrupt its proper functioning. The second stage is a further accumulation of the compound in the cell membrane, leading to a disturbance in the flow of ions through it, which in turn leads to the inhibition of the microorganism growth [75]. In 2018, an attempt was made to precise the mechanism of geraniol action. Geraniol significantly slowed down growth from as little as 0.1% (*v*/*v*). It was confirmed that geraniol induced osmotic stress and DNA damage. Additionally, the researchers found that this compound reduced the levels of its own metabolites, dehydroergosterol (DHE) and H_2_O_2_ in a time-dependent manner [76].

Invasive candidiasis is the most frequent mycosis, causes a high morbidity and unacceptable mortality, especially in critically ill patients, particularly those with immunodeficiency [77]. Geraniol inhibited the growth of *Candida albicans* (MIC = 19.5 mM). This compound caused a reduction in ergosterol levels and altered ATPase activity in the plasma membrane. It causes mitochondrial dysfunction, impaired iron homeostasis. Geraniol also affects the cell wall, but the functional calcineurin pathway appears to be necessary for the antifungal activity of geraniol, since the calcineurin deficit mutant was hypersensitive to geraniol, while the calcineurin overexpressing strain remained resistant to geraniol [78,79].

*Candida albicans* often causes also vaginitis. Geraniol also inhibited the development of biofilm (>80%, at concentration of 0.06%). Washing the vagina with a solution in the concentration of 25μg/mL significantly reduced the number of viable cells [2,80]

Many plant extracts show synergistic activity against microorganisms. The essential oil of *Pelargonium graveolens* and geraniol itself have shown such activity in combination with ketoconazole against *Trichophyton schoenleinii* and *T. soudanense* and in combination with Norfloxacin^®^ against *Bacillus cereus* and *S. aureus* [81]. Geraniol, the active ingredient of the EO of *Helichrysum italicum*, has also significantly increased the effectiveness of *β*-lactam atibiotics, quinoline and chloramphenicol. [2] Geraniol is also the main component (22.33%) of *Thymus glabrescens* Willd (Lamiaceae) essential oil. It showed synergistic effects with chloramphenicol (FIC in the range 0.21–0.87) compared to *E. coli* ATCC 25922, *K. pneumoniae* ATCC 700603, *P. mirabilis* ATCC 12453, *P. aeruginosa* ATCC 27853, lowering the MIC value for this antibiotic, even 10 times [82] It is worth noting that it cannot be assumed in advance that geraniol will have a synergistic effect, because, e.g., in the study against *S. aureus,* MRSA ATCC 25923 geraniol alone reached MIC = 55 mg/mL, but in combination with penicillin it showed an indifferent effect [83].

## 5. Anti-tumor Activity of Geraniol

Geraniol displays anticancer activity against many human cancers in both in vitro and in vivo studies [2]. Although many publications have been published about its anticancer activity, the mechanism of geraniol molecular activity has still not been fully explained [84].

Lung cancer is the main cause of cancer death in the world for both women and men [85]. Galle et al. [86] conducted tests on the human lung adenocarcinoma A549 (ATCC CCL-185™) cell line and additionally implanted A549 cells in naked mice fed a diet with 25, 50 and 75 mmol geraniol/kg. Geraniol caused dose and time dependent inhibition of tumor growth in vivo with the induction of apoptosis. It also influenced cholesterogenesis by the inhibition of 3-hydroxy-3-methylglutaryl-CoA reductase (HMGCR) in A549 cells and decreased serum cholesterol levels in mice with A549 heteroplastoma. Moreover, geraniol lowered the level of expression of membrane-related Ras proteins in hetero-transplanted mice without corresponding changes in level of total Ras proteins. [79,86,87]

In a study conducted by Crespo et al. on the same cell line of lung adenocarcinoma, geraniol stopped A549 cells on the G_0_/G_1_ interphase of the cell cycle and influenced DNA synthesis with a slightly higher sensitivity than HepG2 cells. The researchers also attempted to assess the oxidative status of A549 cells, because oxidative stress induced by chemotherapeutics may be one of the mechanisms responsible for the destruction of cancer cells. Lipids proved to be more sensitive than DNA to oxidative damage caused by geraniol. At 200 μM of geraniol, TBARS levels increased by 122% in A549 cells compared to the control (*p* < 0.05), but no decrease in the activity of such enzymes associated with reactive oxygen species (ROS) biotransformation as superoxide dismutase (SOD), catalase (CAT) and glutathione S-transferase (GST) was observed. Therefore, further studies are necessary to assess more precisely the oxidative status in A549 cells [84].

Colorectal cancer (CRC) is the second most common type of cancer in the world and is one of the main causes of cancer mortality, with approximately 1.8 million new cases worldwide in 2018 [88]. Therefore, this kind of cancer is still a key public health problem [89]. The effect of geraniol on dimethylhydrazine-induced colon carcinogenesis has been studied in a group of 3-week-old male Wistar rats. The study showed that in the chemopreventive action of geraniol no antioxidant activity was observed. Cell death was induced by lowering the level of Bcl-2, the concentration of which increases in the initial phases of carcinogenesis. Apoptosis induction by the Bcl-2 family is one of the main mechanisms of isoprenoid activity also observed in case of perillyl alcohol, geranylogeraniol and farnesol [90].

The activity of geraniol was also studied on the intestinal adenocarcinoma Caco-2 cell line. Geraniol induced the depolarization of plasma membrane potential as a result of the local perforation of the cell membrane with a decrease in membrane resistance in the cells. Additionally, the incubation of Caco-2 cells with geraniol at 400 µM for 6 h resulted in a 60% decrease in protein kinase C (PKC) activity. Additionally, after 16 h of incubation, geraniol caused a 50% decrease in the number of active forms of extracellular protein kinases regulated by p44/p42 (ERC) signal [91]. The inhibition of DNA synthesis and cell cycle arrest in the S phase due to decreased ornithine decarboxylase (ODC) activity were also observed. ODC is responsible for synthesis of polyamines, e.g., putrescine, that also stabilize DNA. Geraniol also caused the accumulation of N-acetylospermidine [92]. It is worth emphasizing that polyamines metabolism is a promising goal of the strategy of developing chemotherapeutic and chemopreventive drugs, so the inhibition of ODC activity may have clinical importance [87,93].

Despite advances in innovative anticancer therapies, 5-fluorouracil (5-FU) is still one of the most effective and most commonly used agents in the treatment of colorectal cancer and it is a major component of many chemotherapy schemes used [94,95]. This compound is an antimethabolite, first synthesized in 1957 [96]. It was interesting to investigate whether this compound in combination with geraniol would show synergistic effects.

Geraniol (150 µM) effectively reduced the levels of thymidine kinase and thymidylate synthase expression. Simultaneous administration of 5-fluorouracyl (20 mg/kg) and geraniol (150 mg/kg) resulted in 53% tumor volume reduction in Swiss nu/nu mice with implanted human TC-118 cancer cells, while a 26% reduction was achieved for geraniol alone and 5-fluorouracyl alone showed no effect [97].

Since vascularization is the main condition for metastasis formation, the anti-angiogenic effects of geraniol have been studied using mice with the dorsal implantation of CT26 colorectal cancer cells. Geraniol inhibited the vascularization of CT26 tumors, which was associated with a smaller tumor size compared to the control. In vitro, geraniol decreased migration activity of eEND2 endothelial cells. Geraniol decreased the level of proliferating cell nuclear antigen (PCNA) and increased caspase-3 expression in eEND2 cells. Moreover, geraniol blocked the signal transduction of vascular endothelial growth factor (VEGF) / VEGFR-2, causing inhibition of AKT and ERK signal pathways [98].

The anticancer effects of geraniol were also studied on human hepatoma HepG2 cells. In studies conducted by Crespo et al., geraniol caused a statistically significant decrease in SOD and CAT activity in HepG2 cells (ATCC HB-8065™). It induced ROS production but not nitric oxide in HepG2 cells, which indicates that ROS were mainly responsible for the resulting oxidative damage. Lactate release increased statistically significantly compared to the control (*p* < 0.001), by 41% and 86% at 200 µM and 800 µM of geraniol, respectively, which suggests that geraniol also affects the glycolytic pathway in HepG2 cells [84].

Shoff et al. found that geraniol inhibits the activity of liver 3-hydroxy-3-methylglutaryl-CoA reductase (HMG-CoA), an enzyme known primarily for its participation in the biosynthesis of cholesterol in mammals, which also participates in the cell cycle. Therefore, its inhibitors stop the cell cycle between the G_1_ and S phases, so they can potentially have an anticancer effect. It is worth noting that the activity of HMG-CoA is eliminated by mevalonates participating in the posttranslational modification of nuclear proteins, but not by nonsterol or other sterols and end products of the mevalonate pathway, such as geraniol [99].

Another study showed that the combination of simvastatin and geraniol synergistically inhibits cholesterol biosynthesis and the proliferation of the same cell line. However, it was also found that low concentrations of simvastatin or geraniol did not inhibit cholesterol synthesis when used separately, but were effective when used in combination. The concentration of geraniol required to inhibit cell proliferation was 100 times higher than the concentration necessary to clearly inhibit cholesterol synthesis [100].

Geraniol alone inhibits HMG-CoA reductase and the incorporation of ^3^H-mevalonolactone into the protein fraction [101]. However, the administration of statins that activate reductase expression prevents geraniol from inhibiting its activity, and this fact explains in part the synergistic effect of low concentrations of both compounds used simultaneously. Exhaustion of the mevalonate pool consequently reduces the pool of farnesyl and other phosphorylated products that are isoprenylated by various cellular proteins, such as small G-proteins, nuclear lamins and γ-subunit of heteromeric G-protein, which are essential for cell growth and proliferation. However, the researchers reserve that other geraniol activities that make up its synergistic effect cannot be excluded. [100]

At least two studies on the chemopreventive effect of geraniol against liver cancer are known. Ong et al. [102] showed a decrease in the number of pathological pre-cancerous lesions in 8-week-old Wistar rats with cancer induced by 2-acetylaminofluorene (2-AAF). The rats were fed geraniol in a dose of 250 mg/kg. Similar studies have also been conducted by Cardozo et al. [103], who observed the same chemopreventive benefits of geraniol administration in male Wistar rats, whose cancer was induced by phenobarbital (PB). It was also found that geraniol suppressed liver carcinogenesis by inhibiting the tumor promotion phase by inducing apoptosis and reducing RhoA—the liver membrane protein [104].

In turn, the available literature describes studies on the influence of geraniol on the development of human pancreatic adenocarcinoma, which were performed on the BXPC-3 cell line [105]. Geraniol caused the contraction of the cell nucleus and condensation of chromosomes, which suggests that it may inhibit the proliferation of cancer cells by inducing their apoptosis. It also increased the inhibitory effect of gemcitabine (2′,2′-difluorodeoxycytidine, dFdC), which is a standard drug for locally advanced and metastatic pancreatic cancer [106]. However, due to the natural or acquired resistance to the drug, only about 20% of patients see a positive therapeutic effect. Cell proliferation was most significant when geraniol was administered 24 h before incubation with gemcitabine [79,105].

The development of skin cancers is favoured by various carcinogenic factors present in the environment, inflammation, UV irradiation and/or tumour promoters. Skin swelling and the induction of epidermis proliferation increase the risk of cancer development. Additionally, the risk of illness development was increased by the infiltration and activation of leukocytes, which can generate reactive oxygen species (ROS) that contribute to the development of cancer [107].

The biochemical, molecular and histological studies conducted so far indicate that geraniol has antioxidant and anti-inflammatory properties. For this reason, it is believed that geraniol, having a strong preventive potential, can protect against oxidative and inflammatory changes caused by 12-o-tetradecanoylphorbol acetate (TPA), which promotes tumor development in the skin [108]. Local administration of this compound activates inflammation by swelling induction, hyperplasia and COX-2 expression in epidermal cells, which regulates the synthesis of prostaglandin E2 (PGE2) from arachidonic acid. Additionally, the study clearly shows that geraniol activates apoptosis in TPA-induced skin cancers, reducing the expression of antiapoptotic Bcl-2 and increasing the expression of proapoptotic Bax protein. [79,107,109]

In a study conducted by Manoharan and Selvan, geraniol showed chemopreventive effects in DMBA-induced (7,12-dimethylbenz[a]anthracene) skin carcinogenesis in albino mice. The oral administration of geraniol at a dose of 250 mg/kg significantly prevented tumor formation, reduced lipid peroxidation and restored antioxidant levels to an almost normal range [110].

Geraniol as a mevalonate pathway inhibitor has shown a dose-dependent effect on melanoma growth in studies conducted on the B16 mouse cell line, increasing cell doubling time. This effect was canceled by the addition of mevalonate and then the cells resumed normal division [111].

Geraniol was also administered to female C57BL mice on a diet (0.65, 6.5 and 65 mmol/kg, respectively) for 14 days before cancer induction and for another 21 days after tumor transplantation. The growth of melanoma was inhibited already at a geraniol dose of 6.5 mmol/kg of food [109,111].

In turn, in the case of studies conducted on the mouse leukemia cell line P388, geraniol increased the time of cell doubling. At high concentrations (> 1.0 mM), it was lethal for P388 cells [99].

Oral cancer often starts as a small, unnoticed white or red lesion or pain anywhere in the mouth. Although there are several histological types, squamous cell carcinoma (SCC) accounts for about 90% of all cancers in the oral cavity. Despite the development of new treatments, the percentage of people with a 5-year survival of this cancer has not improved in the last 4–5 years. It is still at 50% and only if the change was diagnosed at an early stage [112].

Oral squamous cell carcinoma is the eleventh most common malignant cancer in the world and is the leading cause of cancer deaths in South Asia. The disease is characterized by high morbidity and mortality rates (about 405,000 people worldwide each year). The main risk factors for the development of this cancer include alcohol and tobacco use. In the Indian population, oral cancer accounts for 19% of all cancers among men and is the third most common cancer among women [9].

Geraniol administered to rats inhibited the development of 4NQO induced oral cancer. A significant reduction in the number of tumors and their volume was observed, which may result from the inhibitory effect of geraniol on phase I enzymes and thus blocking the bioactivation of 4NQO to 4HAQO, which is carcinogenic [9].

Furthermore, geraniol regulates the expression of phase II enzymes, such as GST, UDP glucuronosyl transferase (UDP-GT) and cytosolic DT-diaphorase (DTD) through a mechanism involving the binding of transcription factor Nrf2 to the antioxidant response element (ARE). The ability of Nrf2 to activate these phase II enzymes is associated with nucleus accumulation. Studies have shown an increase in the concentration of this factor in the nucleus after geraniol administration, which suggests that the chemopreventive effect of monoterpene alcohol is probably caused by the modulation of Nrf2-ARE signaling [9].

Geraniol was also fed to hamsters at a dose of 250 mg/kg of body weight and it completely prevented the formation of tumors in the cheek mucosa of animals treated with DMBA. It increased the expression of Bax protein, caspase-3 and -9, affected the expression of p53 and Bcl-2. Oral administration of geraniol thus prevented deregulation of the expression of cell proliferation markers, inflammation, apoptosis and angiogenesis at the tumor site [112].

Prostate cancer is the most common cancer in men and the second most common cause of death from cancer [85]. High mortality may result from the fact that this cancer may evolve to a form of prostate cancer where its growth will be androgen-independent (AIPC), which in turn often leads to resistance to available chemotherapy schemes [113]. It is worth noting that precisely metastatic prostate cancer often gains the ability to avoid cell death in the absence of androgens, becoming an extremely aggressive cancer [114].

The researchers found that geraniol prevents cell cycle stoppage, activates the apoptosis pathway, and increases the sensitivity of the androgen-independent prostate cancer PC-3 cells to docetaxel. Geraniol effectively reduced the volume of tumor mass (70% compared to control). PC-3 line was first described in 1979 [115], it originated from metastases to the bone and its cells are highly invasive [116]. Geraniol has been shown to inhibit the growth and survival of cancer cells by reducing the expression of E2F8 transcription factor in studies conducted on this PC-3 line. E2F8 transcription factor is involved in cell angiogenesis and polyploidization, and epidemiological analysis has shown that E2F8 levels are elevated in metastatic prostate cancer, which is associated with poor prognosis. The reduction in E2F8 caused by geraniol was sufficient to inhibit cell growth by inducing cell cycle arrest in the G_2_/M phase [79,117].

In subsequent studies conducted in 2012 on the same cell line, it was confirmed that geraniol induces apoptosis [118]. It also induced the process of autophagy, i.e., the degradation of macromolecular components of the cytoplasm, especially long half-life proteins and whole organelles. Thus, autophagy is responsible for maintaining intracellular homeostasis and enables the survival of cells under stress conditions [119].

In most cases, therapy-induced autophagy in cancer cells allows them to survive, promoting the development of the cancer and potentially leading to the occurrence of resistance to treatment, but it can also act as a suppressor of the cancer transformation and by inducing apoptosis increases the effectiveness of treatment [119]

Geraniol also inhibited serine-treonine kinase (AKT) and activated 5′AMP (AMPK) in prostate cancer cells. [114,118] AKT is responsible for the phosphorylation of many proteins associated with the regulation of basic cellular processes, such as transcription, metabolism, apoptosis, proliferation or migration, and, therefore, the dysfunction of its activation is observed in many human diseases, including cancer [120,121].

Protein kinase activated by AMP (AMPK) is the main sensor of metabolic state, acting at both the cellular level and the whole organism, and is responsible for energy homeostasis. Many scientific reports indicate a link between this kinase and carcinogenesis both at the molecular level and at the level of the whole body, but the mechanism of direct participation of AMPK in the aetiology of these disorders is still unknown [122].

Renal cell carcinoma (RCC) is the most common type of kidney malignancy and represents 2–3% of all malignancies in adults, with an upward trend in the number of detected cases [123]. This is the most deadly kidney cancer. Although there are several therapeutic regimens, including surgery, radio- and chemotherapy, they are unfortunately only effective in treating early lesions, which makes RCC one of the most resistant cancers to therapy [124].

One of the causes of this cancer may be contact with nitrilotriacetate (NTA), which is often used as an alternative to phosphates in household and laundry detergents. It can form complexes with metal ions such as Fe^3+^ or Cu^2+^. Iron chelate (Fe-NTA) has a nephrotoxic effect by generating reactive oxygen species such as hydroxyl radical (OH), which cause lipid peroxidation and DNA damage, contributing to the development of acute and subacute necrosis of the renal tubules and thus to the formation of cancer [125].

Treatment with rat geraniol after chronic exposure to Fe-NTA has resulted in a significant decrease in protein and mRNA expressions, such as NFκB, kidney injury molecule-1 (Kim-1), proliferating cell nuclear antigen (PCNA), p53 and a significant increase in caspase-3, -8 and -9, some of which are classic markers of inflammation, proliferation and apoptosis. Geraniol also restored the activity of antioxidant enzymes such as glutathione *S*-transferase, glutathione peroxidase and catalase in rats, which significantly reduced Fe-NTA induced toxicity [87].

The studies suggest that geraniol is a promising candidate for renal carcinogenesis chemoprevention because in the animal model it lowers the level of several tumor biomarkers at both transcriptional and translational levels [125].

## 6. Conclusions

Geraniol is a common component of many essential oils and it can therefore be easily extracted from natural sources. It is not only widely used as a fragrance compound in cosmetic and household products, but it also exhibits a number of biological activities, such as antimicrobiological, antioxidant and anti-inflammatory. Given its low toxicity and high efficacy, it can also potentially be part of a new class of promising therapeutic agents against many dangerous cancers. However, its potential interactions with other biologically active substances require further research. It is also important to confirm the results obtained so far in vitro and in animal models in further clinical trials.

## Figures and Tables

**Table 1:** Plant resources of geraniol.

Plant	Plant Part	Percentage [%]	Ref.
*Aeollanthus myrianthus*	flower	66.00	[5]
*Aframomum citratum*	seed	70.00	[12]
*Boesenbergia pandurata*	rhizome	26.00	[13]
*Campomanesia adamantium*	leaf	18.10	[14]
*Cymbopogon distans*	aerial parts	18.60	[15]
*Cymbopogon nardus*	whole plant	24.20	[16]
*Cymbopogon nardus*	whole plant	40.50	[17]
*Cymbopogon martinni*	whole plant	74.20	[18]
*Cymbopogon martini* var.*martini*	whole plant	61.40	[19]
*Cymbopogon martinii* Stapf.	na	37.39	[20]
*Cymbopogon martinii* var. *motia*	leaf	93.25	[21]
*Cymbopogon martinii* (Roxb.) Wats. var. *martinii*	seed	88.06	[22]
*Cymbopogon winterianus*	whole plant biomass	25.10	[23]
*Cymbopogon winterianus*	leaf	25.50	[8]
*Elettariopsis elan*	leaf, rhizome and root	71.60	[24]
*Neofinetia falcata*	flower	53.00	[25]
*Ocimum basilicum*	inflorescences	18.30	[26]
*Pelargonium graveolens L’Her ex Ait.*	fresh herb	34.60	[27]
*Rosa bourboniana*	flower	15.80	[28]
*Rosa brunonii* Lindl.	flower	19.20	[29]
*Rosa damascena*	flower	29.30	[19]
*Thymus daenensis*	aerial part	37.20	[30]
*Thymus daenensis*	aerial part	75.70	[31]
*Thymus longicaulis*	aerial part	56.80	[32]
*Thymus longicaulis*	leaf, stem and calyx	27.35	[33]
*Thymus pulegioides* (with lemon odor)	whole plant	23.50	[34]
*Thymus tosevii* var. *tosevii*	whole plant	37.80	[35]
